# Introduced birds incompletely replace seed dispersal by a native frugivore

**DOI:** 10.1093/aobpla/plv072

**Published:** 2015-07-02

**Authors:** Liba Pejchar

**Affiliations:** Department of Fish, Wildlife and Conservation Biology, Colorado State University, Fort Collins, CO 80523, USA

**Keywords:** Ecological processes, forest regeneration, frugivory, Hawaiian islands, invasive species, mutualism, plant–animal interactions, seed rain

## Abstract

The widespread loss of native species and the introduction of non-native species has important consequences for island ecosystems. Non-native species may or may not functionally replace the role of native species in ecological processes such as seed dispersal. Although the majority of Hawaii's native plants require bird-mediated seed dispersal, only one native frugivore, Omao (*Myadestes obscurus*), persists in sufficient numbers to fill this functional role. Omao are restricted to less than half their original range, but two introduced frugivores are abundant throughout Hawaii. Given large-scale extinctions on islands, it is important to understand whether introduced birds serve as functional replacements or whether the absence of native frugivores alters plant communities. To assess seed dispersal by native and introduced birds, seed rain, vegetation characteristics, bird diet, density and habitat use were measured at three sites with Omao and three sites without Omao on Hawaii Island. The diet of native and introduced birds overlapped substantially, but Omao dispersed a variety of native species (*n* = 6) relatively evenly. In contrast, introduced birds dispersed an invasive species and fewer native species (*n* = 4), and >90 % of seeds dispersed by introduced birds were from two ubiquitous small-seeded species. Seed rain was significantly greater and more species rich at sites with Omao. These findings suggest that patterns of seed dispersal are altered following the local extinction of a native island frugivore. To more directly evaluate the relative roles of native and introduced frugivores in ecological processes, future studies could include reintroducing Omao to a suitable habitat within its historic range, or novel introductions to nearby islands where closely related species are now extinct. In an era of widespread extinction and invasion of island ecosystems, understanding the consequences of novel animal assemblages for processes like seed dispersal will be critical for maintaining diverse and self-regenerating plant communities.

## Abstract (precis)

In an era of widespread extinction and invasion, understanding the functional role of native and introduced birds in ecological processes is increasingly important. This study compared seed rain in the presence and absence of Hawaii's last abundant native frugivore, and found that introduced birds were an imperfect substitute. In the absence of the native bird, seed rain was less frequent, less species rich, and biased towards smaller seeded plants. To maintain diverse island plant communities, conservation strategies in Hawaii should focus on restoring functional populations of native dispersers, rather than relying on introduced species to fill this role.

## Introduction

Ecosystem processes are increasingly disrupted by the loss and functional extinction of ecologically important species (Anderson *et al*. 2011; Dirzo *et al*. 2014). For example, the decline of fruit-eating birds is altering seed dispersal dynamics (McConkey *et al*. 2012; Kurten 2013), with diverse consequences for plant communities. The absence of vertebrate dispersers can lead to dispersal failure (Chimera and Drake 2010), decrease plant density and diversity (Traveset and Riera 2005; Sharam *et al*. 2009; Traveset *et al*. 2012), shift community composition towards small-seeded generalists and abiotically dispersed plants (Terborgh *et al*. 2008) or a few dominant species (McConkey *et al*. 2012; Kurten 2013) and limit the resilience of plant and animal communities to global change (Elmqvist *et al*. 2003).

In some cases, introduced birds, which are increasingly well established in ecosystems worldwide (Blackburn *et al*. 2009), are the sole dispersers of native plants and may fill the functional role of lost native species (Foster and Robinson 2007), but more often they appear to provide limited benefits or generate negative effects by preferentially dispersing seeds of invasive plants (Kelly *et al*. 2006; Williams 2006; Chimera and Drake 2010; Aslan *et al*. 2012). Understanding the extent to which introduced birds ecologically replace native species is important because seed dispersal is believed to play a crucial role in structuring and maintaining plant communities (Howe and Smallwood 1982; Terborgh *et al*. 2008) through several well-established mechanisms. For example, bird-mediated dispersal enhances recruitment by moving seeds away from parent plants to suitable microsites, allowing seedlings to escape competition and density-dependent mortality (Janzen 1970; Connell 1971; Wotton and Kelly 2011). Thus, if introduced species are inadequate substitutes for native frugivores, the rapid decline and extinction of native frugivores is even more likely to dramatically alter plant assemblages (Bond 1994; Sekercioglu *et al*. 2004; Traveset and Richardson 2006) and reduce overall native plant diversity (Webb and Peart 2001).

The ecological role filled by native frugivores may be disproportionally important on islands such as Hawaii, where most woody plants have historically been dispersed by birds (Carlquist 1974). This relationship between native birds and plants is threatened by the loss or functional extinction of much of Hawaii's avifauna from habitat loss, disease and predation by introduced mammals (Pratt *et al*. 2009). In particular, all crows (*Corvus* spp.) and thrushes (*Myadestes* spp.) are extinct in the wild with the exception of a critically endangered thrush (Puaiohi; *Myadestes palmeri*) on Kauai Island and Omao (*Myadestes obscurus*) on the Island of Hawaii. The Omao is locally common and believed to be stable, but globally listed as vulnerable to extinction (Birdlife International 2012) because it is restricted to a fraction of its historic range (25–30 %) on a single Island (Scott *et al*. 1986; Wakelee and Fancy 1999). Reasons for this range contraction are not well understood (Ralph and Fancy 1994), but could include historic habitat degradation (Scott *et al*. 1986) and fragmentation (Wu *et al*. 2014), predation by introduced mammals (Kilpatrick 2006) or exposure to diseases such as avian pox or malaria (Atkinson *et al*. 2001).

Concurrent with the extinction and decline of Hawaii's native birds, the archipelago has received more introduced birds than anywhere else in the world (>58 species established; Moulton and Pimm 1983; Pyle 2002). Two of these birds, Japanese White-eye (*Zosterops japonicus*) and Red-billed Leiothrix (*Leiothrix lutea*), introduced from east Asia and the Indian subcontinent, respectively, have been highly successful in colonizing native forest communities. Both of these species are small bodied and small gaped (Japanese White-eye: weight 10–12 g, culmen 10–11 mm; Red-billed Leiothrix: 20–23 g, 10–12 mm). Japanese White-eye are generalists that consume insects, fruit and nectar; Red-billed Leiothrix consume fruit as well as insects (Mountainspring and Scott 1985; Male *et al*. 1998). In contrast, Omao are larger-bodied (weight 46–49 g, culmen 12–16 mm), but like Red-billed Leiothrix, they are primarily frugivorous and also consume insects (Ralph and Fancy 1994; Wakelee and Fancy 1999). Each of these species belong to a different family within the order Passeriformes.

Because introduced frugivorous species are abundant throughout Hawaii's forests, in the absence of native frugivores, they have the potential to play a positive role in maintaining native plant communities (Foster and Robinson 2007). Alternatively, Japanese White-eye and Red-billed Leiothrix may only disperse a subset of native seeds dispersed by Omao, or they may facilitate the spread of undesirable invasive plants (Traveset and Richardson 2006).

To address the role of native and introduced birds in seed dispersal and the regeneration of Hawaii's plant communities, this study poses the following specific questions. (i) Do introduced birds disperse the same seeds as the native Omao? (ii) Do landscapes with Omao receive a higher abundance and diversity of seed rain from native plants relative to landscapes without Omao? (iii) Does habitat use by Omao differ from introduced frugivorous birds?

These questions are highly relevant to several emerging topics in applied ecology, including whether or not to embrace novel ecosystems (areas where non-native species are established and inter-mingle with native species), and under what circumstances to pursue refaunation as a conservation strategy. Refaunation is defined as the restoration of ecologically important species either within or beyond their historic range. Making informed decisions about refaunation requires understanding the relationship between native and introduced animal communities and ecosystem processes (Armstrong and Seddon 2008), particularly in the increasing fraction of the world that is dominated by novel ecosystems (Hobbs *et al*. 2009).

## Methods

### Study sites

This study took place from 1 June 2006 to 20 August 2006 and was located on the Island of Hawaii at three sites where Omao are present and three sites where Omao occurred historically but are now locally extinct. The three sites with Omao are the Pua Akala tract of Hakalau Forest National Wildlife Refuge, Keauhou Ranch, which is owned and managed by Kamehameha Schools, and The Nature Conservancy of Hawaii's Kaiholena Preserve. The three sites without Omao are Puu Waawaa Bird Sanctuary, managed by the Hawaii Department of Natural Resources, Honaunau Forest Reserve, owned and managed by Kamehameha Schools, and The Nature Conservancy of Hawaii's Kona Hema Preserve.

The sites with and without Omao are spatially segregated due to the contraction of the former range of this species. All sites, however, can be characterized as mesic rainforest with precipitation ranging from ∼900–2000 mm year^−1^ (Juvik and Juvik 1998). These study sites were located at elevations between 975 and 1900 m **[see Supporting Information]**. The vegetation communities are dominated by ohia (*Metrosideros polymorpha*) and koa (*Acacia koa*) in the canopy and diverse sub-canopy and understory plants, many of which are woody fleshy-fruited species.

Each of these sites is recovering from a varied history of grazing by cattle and other introduced mammals, but management for these species did not vary systematically between sites with and without Omao at the time of the study. The sites with Omao have all experienced a long history of herbivory by cattle and feral pigs (*Sus scrofa*) either within or immediately adjacent to the forest. Feral pigs were present at all three of these sites during the time of this study, and management activities to remove or reduce feral pig densities was occurring at two of the three sites. Similarly, the sites without Omao also have a long history of disturbance by the same non-native mammals, and with the exception of the Kona Hema preserve, all contained feral pigs at the time of this study. Active management to remove feral pigs was occurring at the Puu Waawaa bird sanctuary.

A square grid was established at each site that consisted of 16 points, 200 m apart. All seed rain traps, vegetation surveys and point counts were conducted at these points and all collection of faecal samples and bird habitat use observations occurred within these grids.

### Bird diet

In order to determine whether Omao and introduced frugivorous birds disperse seeds from different sets of plants, faecal samples were collected and analyzed from each target bird species. Faecal samples were obtained by using mistnets to capture birds. Nets (*n* = 6–12) were in use for 3–4 days/month at each site throughout the duration of the field season for a total of 3160 net hours. The nets were opened at approximately 0630 (1 h after sunrise) and closed at 12 pm, or earlier in the case of inclement weather. All potential frugivores [Omao, Red-billed Leiothrix, Japanese White-eye, Hawaii Amakihi (*Hemignathus virens*) and Northern Cardinal (*Cardinalis cardinalis*)] were extracted from the nets and placed in clean cotton bags, at which point the bird would usually defecate. The faecal sample was then removed from the bag, placed in a plastic vial with 70 % ethanol and stored in a cool location. Metal and colour bands were placed on each bird, and weight, wing length, tail length, tarsus length, bill length and sex and age (when possible), were recorded. Other research teams working at the same sites during the same time period, occasionally collected faecal samples from these study species and donated them to this study. All of the faecal samples were later sorted under a light microscope. Seeds were counted and identified to species using a reference collection of seeds collected from the same sites.

### Vegetation

To test for potential differences in plant species diversity and density among sites with and without Omao, per cent cover of all plant species in 10 m radius plots was recorded. Using ocular methods, per cent cover was estimated at three canopy levels at all sites: overstory (>10 m), understory (1–10 m) and ground cover (<1 m). The vegetation plots were located at all odd-numbered grid points at each site, resulting in 8 plots/site or a total surface area of ∼2500 m^2^/site. These data were used to calculate per cent cover (m^2^/ha) of ground cover types, fleshy-fruited plant species and canopy species at each site. Each month of the study (*n* = 3) fruiting phenology was documented by recording the extent to which each individual fleshy-fruited plant species within 10 m of the plot centre was fruiting. The per cent of all surface area of a particular species that was in fruit was estimated using the following categories: 0, 1–33, 34–66 and 67–100 %. Only ripe fruits were included in this estimate.

### Seed rain

To test for differences in seed dispersal by birds at sites with and without Omao, 40 seed traps were established at each site (total traps = 240). Groups of 10 traps were clustered at four randomly chosen grid points. Half of the traps at each point were placed under randomly chosen wind-dispersed canopy trees (*M. polymorpha*) and the remaining half were placed under randomly chosen fleshy-fruited understory plants. Each trap was composed of a 33-inch tomato cage sunk into the ground with a seven gallon plastic plant pot sitting firmly on top of the cage. The funnel-shaped cage was designed to make the traps largely inaccessible to seed predation by rats and mice. Rat faeces were observed in the traps only two times and these faeces may have fallen from the tree canopy. The plant pots had holes on the bottom for drainage and were covered in fine mosquito mesh fitted so that the mesh formed a concave bowl within the pot. A rock was placed on top of the mesh to prevent wind from disturbing the concave/funnel shape of the mesh. All bird-dispersed seeds and other organic material were thus caught within this concave mesh funnel.

Once each month all of the traps were checked, large pieces of litter (e.g. branches) were brushed off above the trap and the remaining material was transferred to a quart size zip-lock bag. The content of these bags were then examined under a light microscope and all seeds were counted and identified to species. Only seeds without a fruit coating were counted. In addition, because decomposition could result in fruit removal from seeds, seeds of the same species as the tree and shrub species above the trap were excluded from the analysis.

### Frugivorous bird density

Point counts were conducted at all six sites to determine the density of each frugivorous bird species. Following standard procedures for 8-min variable circular point counts (Reynolds *et al*. 1980), the following information was recorded: distance to each bird, the method of detection (visual or aural) and several weather parameters (cloud cover, rain, wind). All point counts took place from 0630 to 1100 during June 2006, which is within the breeding season for all of the focal bird species.

### Habitat use

Habitat use by frugivorous birds could influence patterns of seed dispersal. To explore this hypothesis, at least 20 individuals of each focal species were observed at all study sites. Birds were located by systematically traversing each study site and searching for individuals of each focal species (Vanderwerf 1994; Pejchar *et al*. 2005). Observation times varied from 1 to 8 min, and terminated when visual contact with the bird was lost. The plant species in which the bird was foraging, perching or vocalizing was recorded, as well as the total time spent in each canopy or understory species.

### Data analysis

Potential differences in the diet of Omao and introduced birds were assessed by calculating the relative proportion of seed species dispersed across all faecal samples for each bird species. Site-level per cent cover (m^2^/ha) of canopy, understory plants and ground cover and the extent of fruiting (proportion of plant surface area) were calculated by averaging values across the eight vegetation plots at each site. To assess potential differences in plant community composition among sites, Student's *t*-tests (two-tailed, unequal variance) were used to compare per cent cover of each plant species, per cent cover of all fleshy-fruited plants, extent of fruiting and species richness of fleshy-fruited plant species at sites with and without Omao.

Prior to comparing seed rain at sites with and without Omao, seed rain was adjusted to account for differences in the number of seed trap days and the per cent cover and extent of fruiting of bird-dispersed plants at each site. First, seed rain was summed across all traps at each site and divided by 50 to determine seed rain/50 days per site. Second, using the vegetation data collected for each site (described above), seed rain was re-calculated as a function of site-specific per cent cover for species that appeared in seed rain (e.g. the sum of the number of seeds per 50 trap days/% cover of each plant species), and the extent of fruiting (sum of the number of seeds per 50 trap days/proportion of canopy in fruit). The difference in seed rain from all plant species among sites with and without Omao was assessed using a full factorial two-way ANOVA with site type (with or without Omao) and trap location (under fleshy-fruited or wind-dispersed plants) as the main effects and site type × trap location as an interaction effect.

To achieve sufficient replication for statistical analysis, only seed species that occurred in seed rain at two or more sites with and without Omao met the criteria for species-level comparisons. To compare seed rain of these species among sites, Student's *t*-tests were used to compare the mean ± SD seed rain adjusted for vegetation cover at sites with and without Omao. The same test was used to compare species richness of seed rain between sites with and without Omao. This approach, incorporating sources of variation (number of trap days and vegetation cover) into the response variable (seed rain), was adopted because the limited number of data points in this study (*n* = 6) did not provide sufficient degrees of freedom for a statistical model that included habitat and/or site characteristics as covariates.

To compare bird density (birds/ha) at each site, detections were truncated at a radius of 50 m at each point prior to analysis. Student's *t*-tests were used to compare mean (±SD) bird density of all frugivorous birds, introduced frugivorous birds and each frugivorous bird species at sites with and without Omao. The relative proportion of each species in the frugivorous bird community at sites with and without Omao was also calculated (e.g. density of species X/overall bird density). To explore differences in habitat use by Omao and introduced birds, all observations for each bird species were summed across sites. These data were used to calculate the relative time spent by Omao, Japanese White-eye and Red-billed Leiothrix each plant species (e.g. total time observed in plant species X/total observation time).

## Results

### Bird diet

Faecal samples (*n* = 93) were collected from five bird species known to consume fruit (Omao, Japanese White-eye, Red-billed Leiothrix, Hawaii Amakihi and Northern Cardinal. These samples contained a total of 714 seeds from eight plant species. Two of the bird species had few (Hawaii Amakihi = 1) or no (Northern Cardinal = 0) seeds in their faecal samples, suggesting that they are unlikely to be important seed dispersers in this system. Thus, the following analyses focus on the three bird species which do disperse large numbers of seeds: Omao, Japanese White-eye and Red-billed Leiothrix (Table 1).

**Table 1.** The proportion of faecal samples from native and introduced birds that contained seeds, and the richness of native and introduced seeds dispersed by each species.

Bird species	Per cent of samples with seeds	Native seed richness	Introduced seed richness
Omao *Myadestes obscurus* (*n* = 19)	84.2	6	0
Japanese White-eye *Zosterops japonicus* (*n* = 33)	69.7	4	1
Red-billed Leiothrix *Leiothrix lutea* (*n* = 19)	84.2	4	1

The three seed species that occurred most commonly in all faecal samples were *Vaccinium calycinum* (43.8 %), *Rubus hawaiensis* (36.7 %) and *Cheirodendron trigynum* (6.6 %; Table 2). These species were dispersed by all three bird species. The remaining seed species were less common in the faecal samples and were dispersed only by the introduced bird species or only by Omao. *Perrottetia sandwicensis* (0.3 %) and *Rubus rosifolius* (3.9 %), an introduced plant, were dispersed only by the introduced birds. *Ilex anomala* (6.9 %), *Leptecophylla tameiameiae* (0.4 %) and *Psychotria* spp. (1.4 %) were dispersed only by Omao. The large majority of the seeds dispersed by introduced birds were either *R. hawaiensis* or *V. calycinum*; Japanese White-eye = 90.4 %, Red-billed Leiothrix = 92.1 %). In contrast Omao dispersed seeds more evenly (*I. anomala* = 34.0 %, *V. calycinum* = 22.2 %, *C. trigynum* = 20.1 %, *R. hawaiensis* = 14.6 %; Table 2).

**Table 2.** The per cent of seeds from each plant species found in the diet samples of the native bird (Omao), the two introduced birds (Japanese White-eye and Red-billed Leiothrix) and all bird species combined. Seed sizes range from 0.5 to 4 mm in length. * indicates that the bird or plant species is introduced.

Plant species	Seed size—length (mm)	Per cent of diet			
		Omao	Japanese White-eye*	Red-billed Leiothrix*	All bird species
*R. hawaiensis*	3	14.6	85.9	22.7	36.7
*Vaccinium calycinum*	0.5	22.2	4.5	69.4	43.8
*C. trigynum*	4	20.1	6.2	1.8	6.6
*I. anomala*	2	34.0	0	0	6.9
*Styphelia tameiameiae*	3.5	2.1	0	0	0.4
*Psychotria* spp.	3	6.9	0	0	1.4
*P. sandwicensis*	1.2	0	0.6	0.3	0.3
R. rosifolius*	1	0	2.8	5.7	3.9

### Vegetation

There was no difference in the per cent cover of the primary canopy tree (*M. polymorpha*) or any ground cover type (fern, grass, rock, wood, bare ground, introduced herb, moss) between sites with and without Omao, but sites with Omao did have higher canopy cover of *A. koa* **[see Supporting Information]**. There was also no difference in the species richness of fleshy-fruited plant species (sites with Omao = 11.7 ± 0.4; sites without Omao = 12 ± 0.4), the richness of plant species in fruit during the study season (sites with Omao = 7.7 ± 1.5; sites without Omao = 7.7 ± 1.1) or the per cent cover or extent of fruiting in the most common understory plants between sites with and without Omao **[see Supporting Information]**. Three exotic fleshy-fruited plant species (*Passiflora mollisima*, *Rubus argutus* and *R. rosifolius*) were observed at one or more sites both with and without Omao, and two exotic fruiting species (*Passiflora edulis* and *Psidium cattleianum*) were observed only at one or more sites without Omao.

### Seed rain

A total of 1020 seeds from 11 plant species were collected in 240 traps over the collection period (*n* = 12 150 trap days). Just over half of the traps (52.1 %) contained seeds at some point during the study. Four out of the 11 seed species collected in seed traps (*R. hawaiensis*, *V. calycinum*, *C. trigynum* and *I. anomala*) were dispersed in numbers great enough to allow comparisons between sites with and without Omao. The remaining seven species (*Psychotria* spp., *P. sandwicensis*, *Myoporum sandwicense*, *P. mollisima*, *Clermontia* spp., *L. tameiameiae* and *Myrsine* spp.) were collected and/or were present at only one or two sites with or without Omao, which provided insufficient data for statistical analysis.

Overall, sites with Omao had significantly higher species richness of seed dispersed into seed traps (sites with Omao: 5.6 ± 0.5; sites without Omao: 4.3 ± 0.5; *t* = −2.8; df = 4; *P* = 0.04). These sites also had significantly higher numbers of seeds dispersed even after correcting for the per cent cover of understory plants (sites with Omao = 59.2 ± 9.7; sites without Omao = 12.2 ± 11.5; *t* = 5.4; df = 4; *P* = 0.006) and the per cent cover of fleshy-fruited plants at each site (sites with Omao = 232.7 ± 74.3; sites without Omao = 64.3 ± 73.0; *t* = 2.8; df = 4; *P* = 0.05). Native seed species richness was 5.5 ± 0.9 at sites with Omao and 3.3 ± 1.2 at sites without Omao. No exotic species appeared in the seed rain at sites with Omao and only a single exotic species (*P. mollisima*) was observed in the seed rain at sites without Omao.

**Figure 1.**
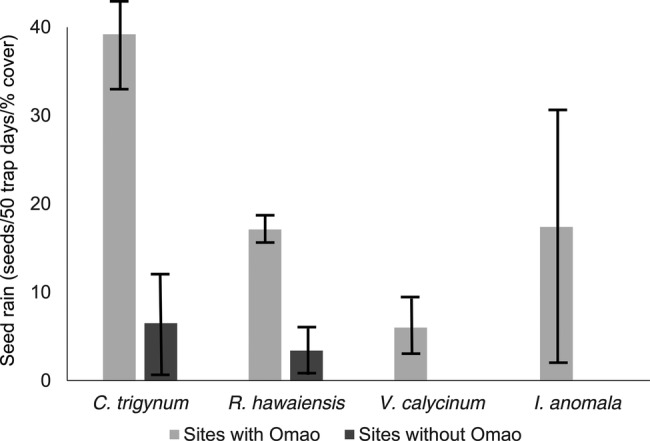
Seed rain from four fleshy-fruited species (number of seeds/50 trap day adjusted for per cent cover of plant species; mean ± SD) at sites with and without Omao.

There was significantly more *R. hawaiiensis* and *C. trigynum* seeds dispersed (relative to per cent cover of each plant species) at sites with Omao (*R. hawaiensis*: *t* = 7.7; df = 3; *P* = 0.004; *C. trigynum*: *t* = 3.5; df = 3; *P* = 0.04; Fig. 1). *Vaccinium calycinum* seeds were only dispersed at sites with Omao; this species was not observed in fruit at non-Omao sites during the study season **[see Supporting Information]**. *Ilex anomala* seeds were only dispersed at the sites with Omao (Fig. 1), despite no difference in *I. anomala* per cent cover or fruiting among the sites with or without Omao **[see Supporting Information]**.

**Figure 2.**
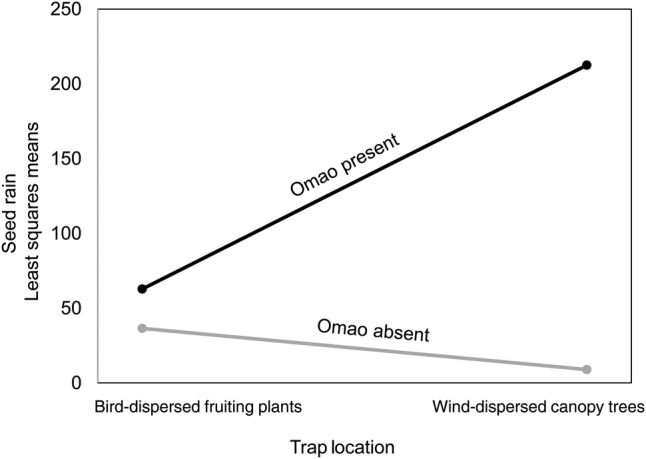
Seed rain (least squares means) under a wind-dispersed canopy tree (*M. polymorpha*) and under fleshy-fruited plants at sites with and without Omao.

The two-way analysis of variance yielded differences in seed rain among sites, trap locations and the interaction between those two main effects (*F*(3,8) = 22.6; *P*< 0.0003). Seed rain differed by site type (sites with and without Omao; *P* < 0.0003), and trap location (under bird-dispersed fleshy-fruited plants or wind-dispersed canopy trees—*M. polymorpha*; *P* < 0.0128). The interaction effect (site type × trap location) was also significant (*P* < 0.0017); more seeds were collected under *M. polymorpha* at sites with Omao (Fig. 2).

### Frugivorous bird density

**Figure 3.**
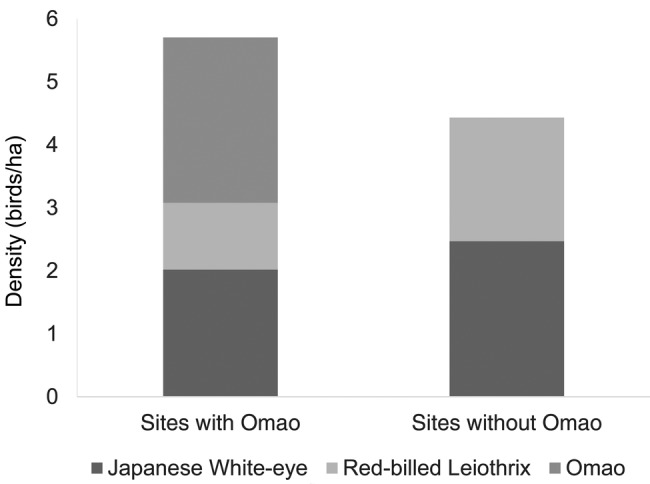
Mean density (birds/ha) of frugivorous bird species at sites with and without Omao.

The density (birds/ha) of introduced frugivores was significantly higher at sites without Omao (sites with Omao = 3.1 ± 0.1; sites without Omao = 4.4 ± 0.8; *t* = −0.3; df = 2; *P* = 0.05), but overall density of frugivores, and density of individual frugivores did not differ between sites with and without Omao **[see Supporting Information]**. On average, Omao consisted of 46 % of the frugivorous bird population at sites with Omao. At these sites, Japanese white-eyes were 35 %, and Red-billed Leiothrix were 19 % of all frugivorous birds. At sites without Omao, Japanese White-eye were 56 % and Red-billed Leiothrix were 44 % of the frugivorous bird population. The absence of Omao from a site was thus associated with a 1.6× increase in Japanese White-eye and a 2.3× increase in Red-billed Leiothrix (Fig. 3).

### Habitat use

The three focal bird species were observed for a total of 535 min [Japanese White-eye 105 min (*n* = 36 individuals); Omao 352 min (*n* = 55 individuals); Red-billed Leiothrix 66 min (*n* = 20 individuals)] throughout the study period. Omao spent most of their time perching and calling from *M. polymorpha* (71 % of total observation time), secondarily on *C. trigynum* (14 %) and sparingly in five other species **[see Supporting Information]**. Japanese White-eye also spent most of their time in *M. polymorpha* (64 % of total observation time), and were observed in 12 other species relatively evenly. In contrast, Red-billed Leiothrix were observed mostly in understory plants. This species spent most time in either *R. hawaiensis* (55 % of total observation time) or *V. calycinum* (29 %), venturing into *M. polymorpha* <5 % of the total observation time **[see Supporting Information]**.

## Discussion

The decline and extinction of native frugivorous birds could have important consequences for plant communities by altering patterns of seed dispersal (Loiselle and Blake 2002; Traveset and Richardson 2006). Alternatively, introduced birds may functionally replace ecological services provided by native species (Foster and Robinson 2007; Aslan *et al*. 2014). Understanding which of these is true is critical to knowing whether to prioritize conservation and refaunation of native dispersers within their historic range, to consider introducing ecologically important species outside their native ranges as proxies for extinct species, or to embrace some introduced species as fulfilling valuable ecological functions. These options are not mutually exclusive and each may be more or less desirable depending on context-specific conservation objectives.

In this exploratory study, Omao and introduced frugivorous birds dispersed similar seed species on Hawaii Island, but in different proportions. Faecal samples of introduced birds were strongly (>90 %) dominated by two common native species, while Omao dispersed a greater variety of seeds in more equal proportions. Only the introduced birds dispersed seeds from an introduced plant. Seed rain was greater and more diverse at sites with Omao, even after accounting for small differences in per cent cover of fruiting plants. Finally, more seeds were dispersed under wind-dispersed canopy trees than understory fleshy-fruited plants at sites with Omao. This finding is consistent with observed patterns of habitat use. Omao spent most of their time in the canopy; in their absence, a greater proportion of the frugivore community was active in the understory. Although this study measured only bird-mediated seed dispersal, this result suggests that not only is seed rain less frequent, less species rich and biased towards smaller seeded plants, but without native frugivores, seeds may also be dispersed into different microclimate conditions because of differences in habitat use among native and introduced frugivores.

In comparison to Omao, both Japanese White-eye and Red-billed Leiothrix are small-bodied generalists. Japanese White-eye in particular have a narrow, piercing bill and may remove flesh from fruit rather than consuming seeds. The smaller gape and more omnivorous diet of the introduced birds together with their diet, as observed in this study and others (Wu *et al*. 2014), suggest that these species are an incomplete ecological substitute for Omao. In contrast to this study, which found substantial dietary overlap between Omao and introduced birds, Wu *et al*. (2014), also working on Hawaii Island but in a single location, found that Japanese White-eye only disperse two seed species. These species were *R. hawaiensis* and *V. calycinum*, which also dominated the diet of introduced birds in this study.

Previous research has suggested introduced birds play an important ecological role in Hawaii, particularly in the absence of native frugivores. Foster and Robinson (2007) studied seed dispersal on the Island of Maui, where all native frugivores are presumed extinct. They reported a very different composition of seeds in faecal samples of introduced birds, despite working in a plant community dominated by similar species as this study. These differences may be partially explained by the length of their field season; they collected faecal samples over a full year, which may have included fruit that was not available or less abundant during the shorter timeframe of this study.

Common to these studies and others (Chimera and Drake 2010) is the concern that introduced birds could exacerbate the spread of invasive species. Foster and Robinson (2007) found that introduced birds disperse *Hedychium gardnerianum* and *R. argutus* and this study found evidence of regular dispersal of *R. rosifolius*—these species are all either considered noxious weeds in Hawaii (USDA 2015) and/or are listed in the IUCN's global invasive species database (IUCN 2015). Wu *et al*. (2014) demonstrated that Japanese White-eye can disperse seeds further than Omao. Collectively, these findings suggest introduced birds may contribute to primary succession, as well as spread small-seeded invasive species, particularly in fragmented landscapes. Additionally, larger seeded plants may be more susceptible to dispersal failure in the absence of the Omao and Hawaii's largest extant frugivore, the Hawaiian Crow (*Corvus hawaiiensis*), which persists only in captivity (Culliney *et al*. 2012).

The decline in the frequency and diversity of seed rain at sites without Omao supports the prediction that the loss of native frugivores alters patterns of seed dispersal. Bird-mediated dispersal, however, is just one component of the maintenance and regeneration of plant communities. Although measuring seedling recruitment and seedling survival was beyond the scope of this study, understanding whether plants are dispersal-limited or constrained by other factors (e.g. competition, seed predation or seedling herbivory by other introduced species) is critical (Denslow *et al*. 2006; Pender *et al*. 2013). Introduced rats, for example, have been shown to interact with native and invasive plants through both seed predation and dispersal (Shiels 2011; Shiels and Drake 2011).

Although the phenology of fruiting did not differ substantially among sites for most species **[see Supporting Information]**, this may have contributed to the seed rain patterns observed, particularly since the sites with and without Omao were spatially disparate. For example, *V. calycinum* was only dispersed at sites with Omao. Although this species was present at all sites, it was not observed fruiting at sites without Omao during the study season. In contrast, *I. anomala* was also only dispersed at sites with Omao, but there was no difference in the per cent cover or extent of fruiting in this plant among site with and without Omao (Fig. 1). Further, because many bird-dispersed species in Hawaii have long fruiting seasons, and not all of these overlapped with the period of this exploratory study, year-round and multi-year studies of the temporal and spatial patterns of bird diet and seed rain are warranted.

In the absence of Omao, the frugivorous bird community appears to shift towards greater dominance of Red-billed Leiothrix. It is unclear whether this change is due to relaxed competition with Omao or due to fundamental differences in site and habitat characteristics that were not measured in this study. Understanding the degree to which introduced species compete with native species is a ripe area for further inquiry in Hawaii and other island ecosystems (Moulton and Pimm 1983). Preliminary observations of habitat use in this study suggest that Red-billed Leiothrix forage largely in the understory and rarely venture into the canopy. This pattern is potentially consistent with the higher seed rain documented under *M. polymorpha* at sites with Omao compared with sites without Omao (Fig. 2). If the gap left empty by Omao is filled disproportionally by a bird that spends time in the understory rather than the canopy, seeds are likely to be dispersed into a different microclimate, which could have important implications for germination success (Janzen 1983). Bird-dispersal that originates in *M. polymorpha* may result in higher germination rates if the seeds are deposited within the canopy (plants that grow epiphytically are protected from introduced mammalian herbivores; Drake and Pratt 2001), or under the canopy, which could shade out introduced grasses and forbs, thus reducing competition for resources (Cordell *et al*. 2009). This prediction, that the absence of Omao leads not only to less seed dispersal overall but also to less seed rain into suitable microhabitat, warrants future research.

In addition to the short time frame and limited observations of habitat use by frugivorous birds, this study has several other important limitations. The sites with and without Omao are necessarily spatially segregated due to the current distribution of this species. Thus, it is possible that factors other than the presence or absence of Omao (e.g. land use history, density of seed predators, patterns of precipitation and primary productivity) could contribute to differences in the rate and magnitude of seed rain and density of introduced birds among the two groups of sites. For example, sites with Omao tend to receive more precipitation than sites where Omao are now absent [**see Supporting Information]**. Despite these limitations, this study's findings are consistent with previous information on the degree of diet specialization of the focal species (Wu *et al*. 2014). Given the restricted range of the Omao and the arguable functional extinction of all other native Hawaiian frugivores (Wakelee and Fancy 1999; Culliney *et al*. 2012), measuring seed rain before and after an experimental reintroduction may be the most effective means of assessing the link between native frugivorous birds, seed rain and plant communities in island systems transformed by anthropogenic activities.

## Conclusions

Introduced birds are imperfect substitutes for native species on Hawaii Island. Although introduced birds provide some seed dispersal services, the relative abundance and proportion of seeds they disperse differs from native species, as does their habitat use, which could have implications for seed germination and seedling survival. Research priorities include understanding which plant species are truly dispersal limited in the absence of native frugivores (Denslow *et al*. 2006; Inman-Narahari *et al*. 2013) and whether these species have shared characteristics on islands with introduced avifauna (e.g. large-seeded plant species; Meehan *et al*. 2002). This requires assessing the importance of reduced or altered seed dispersal services relative to other factors (e.g. seed predation and herbivory) that effect recruitment.

Omao offer a unique opportunity to measure changes in seed rain over time following reintroduction or recolonization. This bird remains common within its current range, and this range may be expanding naturally (Judge *et al*. 2012). If past and ongoing threats are identified and alleviated, active reintroduction of Omao to ensure the persistence of this species and to restore ecological processes may become a priority (Atkinson *et al*. 2001; Fancy *et al*. 2001).

Maintaining the link between plants and seed dispersers is critical on islands such as Hawaii where frugivory is an important ecological process for the majority of woody plants. Given large scale habitat fragmentation and other extinction risks to native frugivores, conserving existing populations should be a priority. Increasingly, island biologists are also considering reintroducing ecologically important species to suitable habitat within their historic range and to neighbouring islands with depauperate communities of vertebrate seed dispersers. Although novel introductions should always be approached with caution, such actions have precedent (Seddon *et al*. 2014) and could offer an exciting opportunity to experimentally evaluate the ecological role of native frugivores. In lieu of handing over the fate of Hawaii's plants to introduced birds, conservation strategies should focus on protecting and recovering native bird species to ensure the maintenance and regeneration of diverse island plant communities.

## Sources of Funding

Funding was provided by Stanford University's Center for Conservation Biology and the Stanford Woods Institute for the Environment.

## Contributions by the Authors

L.P. designed the study, collected the data, conducted the analysis and wrote the manuscript.

## Conflict of Interest Statement

None declared.

## Supporting Information

The following additional information is available in the online version of this article –

**Table S1.** Provides the presence/absence and/or mean (±SD) per cent cover of canopy and understory plant species and ground cover types at sites with and without Omao.

**Table S2.** Provides the presence/absence and/or mean (±SD) extent of fruiting on fleshy-fruited understory plant species at sites with and without Omao.

**Table S3.** Lists the mean (±SD) density of frugivorous birds at sites with and without Omao.

**Table S4.** Reports relative habitat use of canopy and understory plant species by the three frugivorous bird species.

**Table S5.** Reports the elevation and average annual precipitation for each study site.

Additional Information
