# Supplementary material for: Introduced birds incompletely replace seed dispersal by a native frugivore
Source: AoB Plants. 2015 Jul 2;7:plv072. doi: 10.1093/aobpla/plv072 (PMC4526755; doi:10.1093/aobpla/plv072)
Supplement: Additional Information [file supp_7_plv072_index.html]

Introduced birds incompletely replace seed dispersal by a native frugivore — Additional Information 

# Introduced birds incompletely replace seed dispersal by a native frugivore

## Additional Information

Additional Information

- Additional Information - Docx file
