# Supplementary material for: Introduced birds incompletely replace seed dispersal by a native frugivore
Source: AoB Plants. 2015 Jul 2;7:plv072. doi: 10.1093/aobpla/plv072 (PMC4526755; doi:10.1093/aobpla/plv072)
Supplement: Additional Information [file supp_plv072_plv072supp.docx]

SUPPORTING INFORMATION

Table 1. Percent cover of canopy and understory plant species and ground cover at sites with and without Omao. X indicates that the plant species was present, but was observed at too few sites to meet the requirements for statistical analysis. *indicates significance (alpha = 0.05). (E) indicates that the species is introduced to Hawaii.

| Plant species or ground cover | % cover of plant species or ground cover  (Mean +/- SD) | | |
| --- | --- | --- | --- |
|  | Sites with Omao | Sites without Omao | T-test (t, df, p) |
| Canopy trees |  |  |  |
| *Metrosideros polymorpha* | 20.4±11.7 | 7.9±2.8 | t=1.7; df=3; p=0.2 |
| *Acacia koa** | 0.9±1.3 | 13±3.7 | t=-5.3; df=3; p=0.01 |
| Understory fruiting trees and shrubs |  |  |  |
| *Rubus hawaiensis* | 4.5±4.5 | 3.3±2.6 | t=0.4; df=3; p=0.7 |
| *Vaccinium calycinum* | 2.8±1.9 | 0.1±0.1 | t=2.3; df=2; p=0.1 |
| *Cheirodendron trigynum* | 2.8±1.7 | 1.1±1.1 | t=1.4; df=3; p=0.2 |
| *Ilex anomala* | 1.8±1.7 | 1.1±1.6 | t=0.5; df=4; p=0.6 |
| *Coprosma* spp. | 1.6±1.8 | 3.9±2.4 | t=-1.4; df=4; p=0.2 |
| *Alyxia oliviformis* | 0.7±0.5 | 0.2±0.3 | t=1.1; df=3; p=0.3 |
| *Leptecophylla tameiamiae* | X | X | - |
| *Psychotria* spp. | X | - | - |
| *Perrottetia sandwicensis* | X | X | - |
| *Rubus rosifolius* (E) | - | X | - |
| *Passiflora mollisima* (E) | X | X | - |
| *Rubus argutus* (E) | X | - | - |
| *Clermontia* spp. | X | X | - |
| *Cyrtandra* spp. | X | - | - |
| *Pittosporum hosmeri* | X | X | - |
| *Freycinetia arborea* | X | X | - |
| *Broussaisia arguta* | X | - | - |
| *Myrsine* spp. | X | X | - |
| *Hedyotis* spp. | X | X | - |
| *Pipturus albidus* | - | X | - |
| *Myoporum sanwicense* | X | X | - |
| *Fragaria* spp. | - | X | - |
| Richness: | 11.7±4 | 12±4 | t=-0.1; df=4; p=0.9 |
|  |  |  |  |
| *Ground cover* |  |  |  |
| Fern | 19±15.9 | 21.2±8.4 | t=-0.2; df=3; p=0.8 |
| Grass | 43.1±34.4 | 58.8±16.4 | t=-0.7; df=3; p=0.5 |
| Herb | 0.06±0.06 | 1.3±1.1 | t=-1.8; df=2; p=0.2 |
| Rock | 0.6±0.5 | 4.6±6.6 | t=-1.0; df=2; p=0.4 |
| Wood | 3.3±2.5 | 2.1±0.9 | t=0.8; df=3; p=0.5 |
| Moss | 4.5±7.4 | 0.7±0.7 | t=0.9; df=2; p=0.5 |
| Litter | 10.7±10 | 1.2±1.1 | t=1.6; df=2; p=0.2 |

Table 2. Presence and extent of fruiting in understory plant species at sites with and without Omao. X indicates that the plant species was fruiting in at least one site, but was observed at too few sites to meet the requirements for statistical analysis. *indicates a significant difference (alpha = 0.05). (E) indicates that the species is introduced to Hawaii.

| Fruiting plant species | % cover of species in fruit  (Mean +/- SD) | | |
| --- | --- | --- | --- |
|  | Sites with Omao | Sites without Omao | T-test (F, df, p) |
| *Rubus hawaiensis* | 0.3±0.2 | 0.1±0.1 | t=1.0; df=3; p=0.4 |
| *Vaccinium calycinum** | 0.2±0.09 | 0.0±0.0 | t=13.1; df=2; p=0.006 |
| *Cheirodendron trigynum* | 0.4±0.3 | 0.1±0.1 | t=1.3; df=3; p=0.3 |
| *Ilex anomala* | 0.2±0.09 | 0.4±0.3 | t=-1.0; df=2; p=0.4 |
| *Passiflora mollisima* (E) | - | X |  |
| *Rubus argutus* (E) | X | - |  |
| *Clermontia* spp. | - | X |  |
| *Pittosporum hosmeri* | X | X |  |
| *Myrsine* spp. | X | X |  |
| *Alyxia oliviformis* | X | - |  |
| *Hedyotis* spp. | X | X |  |
| *Myoporum sandwicense* | X | X |  |
| *Perrottetia sandwicensis* | X | X |  |
| *Leptecophylla tameiamiae* | X | X |  |
| *Fragaria* spp. | - | X |  |
| *Coprosma* spp. | X | X |  |
| *Rubus rosifolius* (E) | - | X | - |
| *Psychotria* spp. | X | - |  |
| Richness: | 7.7±1.5 | 7.7±1.1 | t=0; df=4; p=1.0 |

Table 3. Density of birds/ha at sites with and without Omao. *indicates a significant difference (alpha = 0.05).

|  | Birds/ha (Mean ± SD) | | |
| --- | --- | --- | --- |
| Bird species | Sites with Omao | Sites without Omao | T-test (t, df, p) |
| Omao | 2.6±1.9 | - | - |
| Japanese White-eye | 2.0±0.5 | 2.5±0.3 | t=-1.4; df=4; p=0.2 |
| Red-billed Leiothrix | 1.1±0.5 | 1.9±1.1 | t=-1.2; df=3; p=0.3 |
| Introduced species* | 3.1±0.1 | 4.4±0.8 | t=-0.3; df=2; p=0.05 |
| All species | 5.7±1.9 | 4.4±0.8 | t=1.1; df=3; p=0.3 |

Table 4. Percentage of time each frugivorous bird species was observed in canopy trees (i.e. *Metrosideros polymorpha* and *Acacia koa*) relative to subcanopy and understory fruiting species.

| Plant species | Omao | Japanese White-eye | Red-billed Leiothrix |
| --- | --- | --- | --- |
| *Metrosideros polymorpha* | 71.2 | 64.4 | 0.3 |
| *Cheirodendron trigynum* | 14.6 | 2.9 | 2.9 |
| *Acacia koa* | 4.7 | 11.7 | 2.9 |
| *Ilex anomala* | 4.2 | 1.2 | 2.7 |
| *Myoporum sandwicense* | 2.8 | 5.7 | 2.4 |
| *Rubus hawaiensis* | 1.9 | 5.7 | 54.8 |
| *Myrsine* spp. | 0.5 | 0.5 | - |
| *Pittosporum hosmeri* | - | 3.2 | - |
| *Scaevola* spp. | - | 1.0 | 0.4 |
| *Coprosma* spp. | - | 0.8 | - |
| *Vaccinium calycinum* | - | 0.7 | 29.4 |
| *Broussaisia arguta* | - | 0.6 | 0.8 |
| *Leptecophylla tameiamiae* | - | - | 3.2 |
| *Clermontia* spp. | - | - | 0.2 |

Table 5. Elevation (m) and annual precipation (mm) of study sites with and without Omao.

| **Sites with Omao** | Elevation (m) | Precipitation (mm) |
| --- | --- | --- |
| Hakalau Forest NWR | 1900 | 2000 |
| Kaiholena Preserve | 975 | 2000 |
| Keauhou Ranch | 1720 | 1900 |
| **Sites without Omao** |  |  |
| Kona Hema Preserve | 1350 | 900 |
| Puu Waawaa Bird Sanctuary | 1524 | 1186 |
| Honaunau Forest Reserve | 1585 | 1219 |
